# An Integrated Multilevel Mental Health Support System for University Students: 4-Year Longitudinal Observational Study

**DOI:** 10.2196/67089

**Published:** 2026-01-26

**Authors:** Szilvia Vincze, Antal Bugán, Karolina Kósa, Zoltán Bács

**Affiliations:** 1 Chancellery University of Debrecen Debrecen Hungary

**Keywords:** mental health, digital health services, college students, student, university, higher education, undergraduate, counselling, young adult, young adults, health-seeking, mood, mHealth, mobile health, app, digital health, smartphone, eHealth, telehealth

## Abstract

**Background:**

University students, in the life stage of emerging adulthood, struggle with a number of mental health problems around the world, mostly due to difficulties related to their studies and social relations. Though most students are aware of their problems, health-seeking behavior tends to lag behind. The COVID-19 pandemic aggravated mental health problems among students. In response, the University of Debrecen developed an integrated, multilevel model system aimed at the screening, prevention, and treatment of students’ mental health problems.

**Objective:**

This paper describes the testing of the integrated, multilevel model system aimed at the screening, prevention, and treatment of students’ mental health problems.

**Methods:**

The new system consists of data collection and intervention or service functions with 2 levels of informal digital, and 3 with partly digital, partly personal service modalities. Students access the system through a dedicated smartphone app that offers other university-related functions requiring personal login. One function of the app involves a mood report with 3 categories (awful, acceptable, and good), of which one per day can be submitted by students. Based on this report, further services are offered. According to the weekly patterns of the mood report, responding students may be directed to the second level, at which screening for depression or willingness to participate in peer groups is assessed. Depending on the responses, students are referred to personal (face-to-face) services at the secondary level for intervention. Aggregated reporting for leadership on the use of functions is available at all levels, which can be used to make decisions regarding the expansion of services or creating new ones.

**Results:**

The model was launched in September 2020 and was tested for 45 months. After an initial increase in use, approximately 29% (8673/29,045) of all students provided mood reports (the University of Debrecen student population on October 15, 2024, was 29,045 students; the student population using the mobile app mood report was 8673 students). The percentage of students reporting a bad mood varied between 8.9% (26,465/297,372) and 12.2% (36,280/297,372) in the test period, while 50% (151,548/297,372 reports) of students reported being in a good mood. There was a marked pattern of increased use of mood reporting during the fall and spring study periods, while usage prominently decreased during the examination period and summer recess.

**Conclusions:**

The 4-year trial period demonstrated that the mood report embedded in the mobile app can identify students with a potentially increased risk of mental health problems in need of support without stigmatization. The unique feature of our model seems to be its app-based screening at the first level and its hierarchy integrating digital and personal services. The system provides an easy-to-follow path from digital to personal services, along with time-specific data on the mental health of students for university leadership to facilitate the development of new services.

## Introduction

University students represent a particular social group of youth that may be considered privileged since they are on their way to becoming part of the educational minority with the highest educational attainment. They are also an educational minority in their own age group, as they pursue tertiary education. 41% of 20- to 24-year-olds in Organization for Economic Co-operation and Development (OECD) countries and 35% in Hungary pursued university studies in 2020 [1].

Despite being young, university students face a range of mental health conditions. According to a survey conducted in the United States in 2001 and 2002, almost half of college-age youth had a psychiatric diagnosis; 11.9% had some degree of anxiety disorder, but less than 25% of those with a diagnosis received treatment [2]. The risk of depression was also found to be a significant problem with a prevalence of 17.3% among US college students [3]. In addition to anxiety and depressive conditions, substance use also represents a considerable problem, and although there are effective pharmacological and other methods to treat all of these conditions, the pattern of help-seeking in this group was below the level of need [4]. Studying outside their home country further increases the risk of stress [5].

A significant number of problems associated with young people in higher education have also been experienced by their peers outside the educational system. This specific period was defined by Arnett [6] as that of “emerging adulthood,” a period of life that marks the transition from adolescence to adulthood with or without the period spent in higher education. Problems of this time of life inspired extensive further research since the beginning of the 21st century, and the World Mental Health Surveys [7], coordinated by the World Health Organization (WHO), established an international network to assess the mental health of students in higher education. Its first survey, conducted between 2014 and 2017 using a standardized methodology, involved students from 19 higher education institutions in 8 countries. According to the results, 35% of respondents had at least one mental disorder at some point in their lives, and 31% had at least one of the most common mental disorders (depression, mania, anxiety disorder, panic disorder, alcohol abuse, and drug abuse) in the preceding year [8]. However, despite common mental health problems, just under a quarter of respondents (24.6%) said they would seek help; they preferred to solve their problems alone or with friends and relatives [9].

The COVID-19 pandemic only worsened the mental situation of students. A survey including students from 41 European countries commissioned by the European Students’ Union (ESU) in April and May 2020 found that 12.9% of respondents had a mental health problem, their emotional well-being had declined, and 7% of students had no one to turn to for emotional support [10]. A large-scale online survey of 350,000 students at 373 universities in the United States, carried out in 2020-2021 using standard screening questionnaires, found that more than 60% of students were identified as having one or more mental health problems, an increase of nearly 50% compared to 2013. The prevalence of various problems and patterns of health-service use also varied across students of different racial groups by self-definition, but help-seeking remained below need in all groups [11]. A follow-up study found that the proportion of students at high risk of anxiety and/or depression remained unchanged from fall 2020 to spring 2021 [12].

The latest results of a survey of UK undergraduates show that while 6% of students in 2017 had a mental health problem, this figure almost tripled to 16% by 2023. The same survey found that 29% of students considered dropping out, and of the 18 factors mentioned as potential reasons, mental health was by far the most frequently selected by the respondents [13].

A German survey completed in 2020 found that 24.7% of students always or almost always felt depressed, and 31% thought they were isolated from their peers.

The unfavorable impact of the pandemic on the mental health of college students was also confirmed in Australia [14], China [15], Bangladesh [16], and India [17].

Similar patterns were identified among Hungarian students before the pandemic. One-fifth of medical students at the University of Debrecen in Hungary had abnormal levels of psychological distress assessed by the 12-item General Health Questionnaire (GHQ-12). Using the same tool, it was shown that students in teacher training in 6 Hungarian higher education institutions were significantly worse off than the same age group of the general population in the first decade of the 21st century [18]. Using the 9-item version of the Beck Depression Inventory (BDI), Lisznyai et al [19] found that depression scores were significantly higher among students completing a master’s degree; higher levels of anxiety were found among 18- to 19- and 24- to 26-year-olds using the STAI questionnaire.

Hungarian research on the impact of the pandemic provided contradictory results. Researchers using a validated mental health tool (Mental Health Test) [20] at the largest medical university in Hungary found no difference in student performance compared to the national average in April 2021[21].

The Association for Counselling in Higher Education (ACHE) invited students from all Hungarian higher education institutions to participate in an online survey in the fall of 2021, in which more than 10,000 students from 47 institutions participated. A total of 10.2% of respondents had been prescribed medication for a mental health problem, of whom 6.2% were also taking medication at the time of responding. The current mental status was assessed by a 45-item screening questionnaire (OQ-45), the mean of which in the Hungarian sample exceeded the clinical threshold established in US studies. According to the shortened version of the BDI, 36% of respondents were at risk of mild and 5.2% of moderate depression; 6.6% of respondents often had suicidal thoughts, 3.4% had those almost always [22]. In total, 70% of respondents were negatively affected by the pandemic, but there was no discussion of how the pandemic changed mental health compared to previous years.

The ACHE carried out a new study in November 2023 involving more than 15,000 students from 43 higher education institutions in Hungary. Furthermore, 44.6% of respondents had moderate or moderately severe depressive symptoms, 16.3% had severe depressive symptoms; 36% often or always felt lonely; 41.4% had a crisis that negatively affected their mental state at the time of the survey, and 3.7% of students had a mental illness at the time of responding [23].

Youth in higher education face significant mental health challenges for complex reasons. The main causes are related to their studies, the extent of knowledge to be acquired in their discipline, which is associated with high expectations, and the time spent on studying. The duration of learning has a positive impact on academic progress but a negative impact on other activities. Academic expectations, including examinations, are a significant part of the stress load for students [24]. Insufficient previous knowledge of the chosen profession or discipline can also be a risk factor that may lead to a decrease or lack of motivation [25]. Other well-known risk factors include insufficient peer support and subsequent social isolation, which may also be related to family problems; conflicts with parents or lack of friends, while a low socioeconomic status and/or financial difficulties may cause livelihood problems. Part-time work can alleviate financial issues, but at the cost of time available for learning. A common risk factor is the overuse of psychoactive substances, partly related to recreational social situations and to the misconception that this can be a solution to mental difficulties [24]. Age, gender, sexual orientation, gender identity, and ethnicity also affect students’ mental health [26], as does any preexisting mental disorder [8,23].

Despite their problems, college students do not take full advantage of support services available due to reasons summarized in a recent systematic analysis of 44 publications [27]. The proportion of students who used any mental health services varied between 13.7% and 68.6%, and between 2.6% and 75% when outpatient service use was estimated. This significant variability could not be explained by the type of services but was related to the size of the at-risk population: a higher number of students with mental health problems was related to a higher rate of service use. The lowest rates of service use were found among students who studied outside their home country. Based on the aggregated data from the articles included in the review, 21% of students used mental health services (95% CI 3%-72%).

Data on mental health service use in Hungary were collected in ACHE surveys. These studies found that 4.4% of students used psychological support services provided by higher education institutions in 2021 [22], and this increased to 7.4% in 2023 [23].

Overall, the mental health of students in higher education institutions is a growing concern across countries and institutions, albeit to varying degrees. Many institutions of tertiary education make considerable efforts to improve the overall health of their students, including mental health, as recommended by the Health Promoting University concept of the WHO [28], based on which specific projects are launched [29].

Digital services offer one type of solution for the reduction of the gap between health care needs and supportive care and services. Based on a review of 89 relevant studies published in 2019, US authors found that 80% of digital interventions were delivered through an internet portal, and a third of these included human contact and support. The majority of programs were either fully or partially effective in achieving positive change in the psychological outcome variables identified for evaluation [30]. A meta-review of 7 meta-analyses published in 2020 found equivocal results in terms of quality of evidence, and small to medium effects for randomized controlled trials investigating stand-alone applications and apps with guidance. It is worth noting that most RCTs included less than 1000 participants, the highest number in the intervention group being 3639 persons [31].

The effectiveness of delivering interventions to improve mental well-being via social media was investigated by Plackett et al [32] in a systematic review based on relevant papers published between 2004 and 2022. Improvements were shown in 39% of the studies, but the rest of the papers—the great majority being of poor quality—found mixed effects or none at all [32]. On the other hand, mindfulness meditation helped by mobile devices, including smartphones and apps, was found to be more effective than control groups in decreasing stress and anxiety [33].

A recent systematic review of 6 studies on treatment apps, 4 studies on self-monitoring apps, and 21 papers on multipurpose smartphone apps for mental disorders found that they were acceptable and effective, but their use declined over time. The authors specifically recommended, among others, that apps provide feedback to patients and clinicians, and even be integrated with patient electronic health records [34].

There are a number of other digital modalities that show promising results among youngsters such as digital platform with chatbot for schoolchildren that helps improve social-emotional learning and self-help [35], smartphone app for helping delinquent adolescents to be more aware of their emotions [36], and family-based telehealth interventions for youngsters in foster care to facilitate their integration [37], but these modalities have not yet been tested among college students.

The University of Debrecen developed a mobile app (Studyversity) to help its students navigate their student life. It can be downloaded from Google Play and Apple Play in both English and Hungarian and is compatible with all mobile platforms. It has a simple graphical user interface design with separate items for each available function. The app contains class schedules of enrolled courses, registered examinations, and individualized academic data such as examination results or financial balances. Class schedules and examination dates are synchronised with the phone’s calendar. A location guide is available to help students find appropriate classroom buildings. Information about academic divisions and their office hours is updated in real time based on information from the Registrar’s Office, along with information on available scholarships. Another function of the app is the University of Debrecen Community Space, which allows students to find companions for various leisure activities—from dog walking to theatre visits. Students are instantly informed about university news and important updates through push notifications, even without launching the app.

All Hungarian universities are legally obliged to protect and promote the mental health of their students. The University, by virtue of monitoring the mental health of its students [25] and maintaining various personal helping services for them that contribute to this legal obligation, acquired experience with such systems. Based on these precedents, the leadership of the University of Debrecen, together with its Students’ Union, decided to expand the Studyversity app with a “mood report” function using a 3-item visual analog smiley face scale to report mood (awful, alright, and great). Its use is incentivized by the opportunity to win university-branded merchandise after submitting a certain number of reports. The mood report was integrated into a multilevel approach to the prevention and treatment of mental health problems among students.

The concept of the system was taken from the foundational concepts for the delivery of mental health care services as described by the WHO [38], along with experiences with the aforementioned services. This paper aims to present the composition and levels of this new service model, investigate the feasibility of its operation, and present data about the use of the digital levels obtained during the test period of 45 months.

## Methods

### Description of the Integrated Multilevel Model of Mental Health Services

The identification of students with mental health needs and finding a way to engage them to seek help while minimizing stigmatization represents a key challenge. Since nowadays each student has a mobile phone that they consider an indispensable tool in their lives, it was decided that a dedicated smartphone app would be used at the informal digital level in a hierarchy of services. The next, also digital level would be designed to engage those who are at a high risk in terms of mental health problems. In terms of screening for depression, the most frequent mental health problem among students, the scientific consensus on screening principles was taken into account [39].

Information for faculty leadership about the number of students in need of help could be provided, and resource-intensive, that is, in-person services would be offered starting at the level of formal health care services. Reporting on the use of services to the leadership of the university would enable them to expand existing or develop new services and interventions should such a need arise. Table 1 provides an overview of all (existing and newly developed) informal and formal levels of the system, along with the means of data collection, provision of interventions and services, and reporting to leadership.

**Table 1:** Organizational levels of providing mental health services at the University of Debrecen.

Type, level according to WHO^a^, and how students can access it				Available service		Data collection				Reporting to leadership^b,c^		
						Description		Type		Description		Type
**Informal**												
	**Self-care**											
		Dedicated online platform	Written, audio, and video materials related to mental health.		Number of users. Time spent at the platform.		Digital		Aggregated data		Table	
	**Community care**											
		Dedicated online platform	Individual consultation (online or in-person), peer groups for study and support (in-person).		Number of consultations. Number of groups. Number of sessions.		Analog		Aggregated data		Table	
	**Self-care**											
		Mobile app	Mood report (3-item visual analog scale with smiley faces).		Number of mood reports. Number of “awful” moods if the number of “awful” moods = 10 in 10 days.		Digital Digital		Aggregated data Aggregated data		Table Table	
**Formal**												
	**Primary**											
		Mobile app	If the risk of depression is elevated, referral to the Centre of Clinical Psychology (CCP).		Screening test offered for depression.		Digital		Aggregated data		Dashboard	
		Primary care physician	Primary care (in-person), if needed: referral to CCP.		Health care system		Digital		Aggregated data		Table	
	**Secondary**											
		Appointment by phone or email	Individual psychological service (outpatient and in-person, referral-based).		Health care system		Digital		Aggregated data		Dashboard	
		Referral	Group psychological service (outpatient and in-person).		Health care system		Digital		—^d^		—	
	**Tertiary**											
		Referral	Psychiatric service (inpatient and in-person).		Health care system		Digital		Aggregated data		Dashboard	

^a^WHO: World Health Organization.

^b^Availability of the reports is guided by the right of access to data as specified in the University by-laws.

^c^New functions developed within the Studyversity app are marked with an asterisk.

^d^Not applicable.

Transitions between digital and in-person services are implemented through structured referral pathways across levels of care.

### Digital Mental Health Services

Our service model was implemented by creating a mobile app for students called UD Studyversity (UDSV). The first version of the UDSV was available for download in September 2020. The free app, available in both Hungarian and English, provided information about a range of student services (educational, administrative, etc.) available at the university as well as a number of functions that the university developed in-house to meet the needs of students (eg, community space for incoming students, display of educational data). Some functions of the UDSV (mostly information also available online on the university website) can be accessed without logging in, while other functions are available after authentication using their unique student ID.

The first new element of the model of mental health care at the informal level is a so-called “mood report,” one of the personalized functions of UDSV that helps identify students at risk for mental health problems. After an authenticated login, students can report their current mood at any time but only once per day by selecting one of three categories (“awful” is bad, “alright” is acceptable, and “great” is good). After sending the mood report, the student receives a positive message to acknowledge receipt of the report in the form of a motivational quote or a short entertaining video, which is not related to the mood report, only rewards the fact that the student submitted it. At the suggestion of the SU, one more feature was added to the mood report to encourage the use of UDSV: points can be earned by those who report their mood consistently every day. Students earning different scores can receive gifts, tickets to university events, or free and discounted university services (sports, meals, etc). These rewards are preselected for each level (score).

Based on consistently submitted mood reports, it is possible to identify students at risk at the informal level. Specifically, students who reported an “awful” mood on a preset number of consecutive days receive a validated online questionnaire to screen for depression, in which the student also gives permission for being contacted by clinical psychologists of the university for further services if his or her screening score reflects an increased risk.

Students who submit “great” mood reports at the informal level on a predefined number of consecutive days receive a questionnaire in which they can declare whether they would like to participate in mentoring peers to help them manage university life in a group setting.

### Personal Mental Health Services

#### Individual Care

Students identified as high risk based on the mood reports may be contacted by clinical psychologists at the formal level, as described above, to offer further personal care. This form of care may take the form of counselling or psychotherapy. Since both of these interventions entail more than one session, it is only appropriate for high-risk students who may be clinically depressed, in crisis, or at risk of suicide. Those in need of this type of care should be diagnosed and treated in person by clinical psychologists or psychotherapists working in the Clinical Center of the university.

#### Group Care

Students who are identified as at risk by the mood report function of UDSV and screened by the questionnaire are offered—depending on the severity of their condition—the opportunity to join groups of 8-12 students. These groups would also include 3-4 students per group who submitted positive mood reports and signed up to be volunteer peer supporters. Groups are organized 4 times a month for 3-hour sessions. These groups serve multiple purposes, such as the provision of peer support and “healthy” feedback from peers, to increase self-awareness and identify common problems and find helpful solutions.

Secondary and tertiary health care are the highest organizational levels at which the leadership of the university would be provided with summary data about the mental health needs of students, along with provided and accessed services, in order to facilitate systemic evaluation and interventions.

#### Promotion, Data Collection, and Statistical Analysis in the Test Period

The UDSV was introduced in September 2020 and was actively promoted among students until the end of the fall semester (mid-December, altogether 4 months). Subsequently, there was no active promotion. The reward system was restructured in February 2024, after which the app was promoted for one month. The test period ended in May 2024, after 45 months.

As it was described above, mood reports can be filled out and submitted after authentication with a unique student ID and password. No special registration is required to use the application itself. With the consent of students, the mood reports are uploaded into the electronic database of the Management Information System (MIS) of the University of Debrecen. The database collecting the mood reports stores the unique student ID, the faculty at which they study, the mood category, and the date and time of the report.

The aggregated data is processed in Microsoft Power BI and used to create summary dashboards for management. There is an interface between the application and the Power BI to ensure an up-to-date data flow. After data processing, various summary evaluations can be created and displayed as dashboards, such as the number of users, the number of mood reports, their categorical distribution by day or by longer time intervals (eg, study period and examination period), or as time-series charts. University and faculty leaders have access to summary dashboards according to their level of authorization.

### Ethical Considerations

The Hungarian Act on Higher Education (Act CCIV of 2011) [40] obliges all institutions of higher education to provide information and consultation for students to facilitate their progress and completion of studies, particulary those with disabilities; and institutions must also provide opportunities and services for promoting health while legal obligations related to data protection and information freedom must also be fulfilled [41]. Therefore, the newly created mobile app and digital screening test are completely separate from the digital educational system of the University, the use of which is mandatory for the students to monitor their educational performance. The application by which mood reports are collected was developed by the University of Debrecen, is the property of the University, and is available only to its registered students. All students of the University who can use smartphones and computers can access the system for the designated purposes. Students with special needs are served by another center of the University that has been established and operated specifically for these students. In order to enter the app, students have to supply their unique student ID, by which they consent to their data being collected by the university. A statement about this must be read and approved by the students upon entering the app. However, the self-reporting of mood and filling out the depression screening test within the app are voluntary, and this is detailed in a statement that must be approved upon entering. Students are not compensated for the use of the app, but small tokens (eg, a t-shirt, paper notes, a pen with the logo of the University) are offered to those who regularly report their mood for at least ten weeks. Participation in the mood reporting and screening functions was entirely voluntary and not linked to any academic requirements or benefits.

The app and digital screening test are parts of the USDV system that is operated by a division independent from any faculty, reporting directly to the Chancellor of the University. Data accessibility for staff members in this division is guided by a written protocol. All persons who can access data of this system bear criminal culpability for misuse of data.

The Regional and Institutional Ethics Committee of the University of Debrecen (2./2025 RKEB), after reading the present manuscript, issued a retrospective Institutional Review Board exemption for this study.

The full database of the system is only available to the head manager of the system and the chancellor of the University. Summary data from the system can be requested in writing by persons in leadership positions (deans of faculties, vice-rectors, and rector) of the university from the head manager. Summary reports are offered in dashboard format with no backtracking to individuals. Others requesting data must submit a research plan specifying the purpose and types of data that are requested. The plan must be approved by the head manager and the chancellor. Upon approval of the plan, deidentified data are provided, and the final results of the analysis must be submitted to the chancellor.

## Results

After the development of the model and the alpha-test phase of the application (mood report and reward system), pilot testing was conducted between September 1, 2020, and May 31, 2024 (45 months), during which time the mood report function was used by 8673 students who provided approximately 297372 mood reports. According to Google Analytics, the mood report was the second most used feature of UDSV, only surpassed by the timetable function linked to the educational administrative system of the University.

The percentage of users of the mood report function out of all students somewhat decreased from almost 8% (2291/29,045 users) during the introductory first 4 months to 6% (1693/28,418 users) in the first full year, but subsequently almost doubled and remained at around 11% (3146/29,021 users) of the active students in the test period (Table 2).

**Figure 1:**
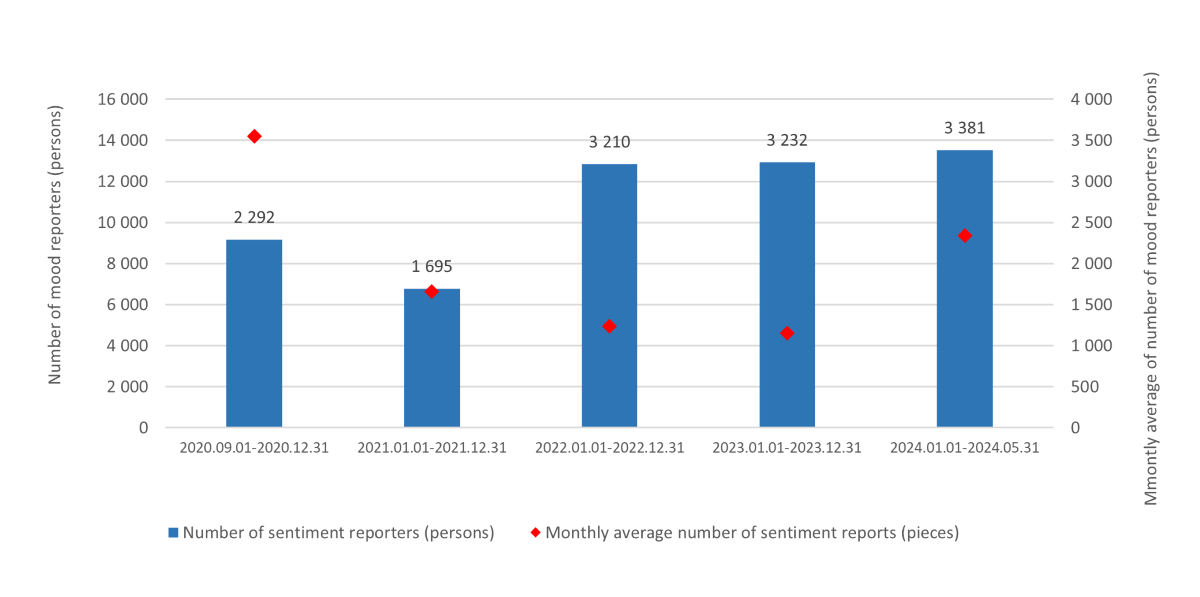
Number of mood reporters and mood reports collected by the smartphone app in the specified time intervals during the test period of the system.

Figure 1 shows a 1.47-fold increase in the number of mood reporters compared to all students and a 35% decrease in the number of submitted reports during the entire length of the test period. The reward system was restructured in February 2024, after which user numbers increased significantly, as seen in Figure 1.

**Table 2:** The percentage of students compared to all registered students who reported their mood by the smartphone app in the specified time intervals during the test period of the system.

Characteristic	2020: September 1 to December 31	2021: January 1 to December 31	2022: January 1 to December 31	2023: January 1 to December 31	2024: January 1 to May 31
Months, n	4	12	12	12	5
UD^a^ students, n	29,045	28,418	28,414	29,021	28,244
Students who submitted mood reports out of all students, n (%)	2292 (7.9)	1695 (6)	3210 (11.3)	3232 (11.1)	3381 (12)

^a^UD: University of Debrecen.

We also analyzed the use of mood reports in the specific study periods of the academic year, that is, during the fall and spring semesters, as well as the winter and summer examination periods, since most functions of UDSV are more useful during the semesters with higher expected usage. At the same time, examination periods tend to be more stressful for students with a negative or wavering impact on their mood.

After the launch of the UDSV until the end of the promotion period, the number of users of the feature as a percentage of the active students exceeded 10% (3382/33,820 users). In the following year, when the app was not promoted, the number of UDSV users more than halved. From 2024, with the promotion of the app that included new functions, UDSV use increased again.

**Figure 2:**
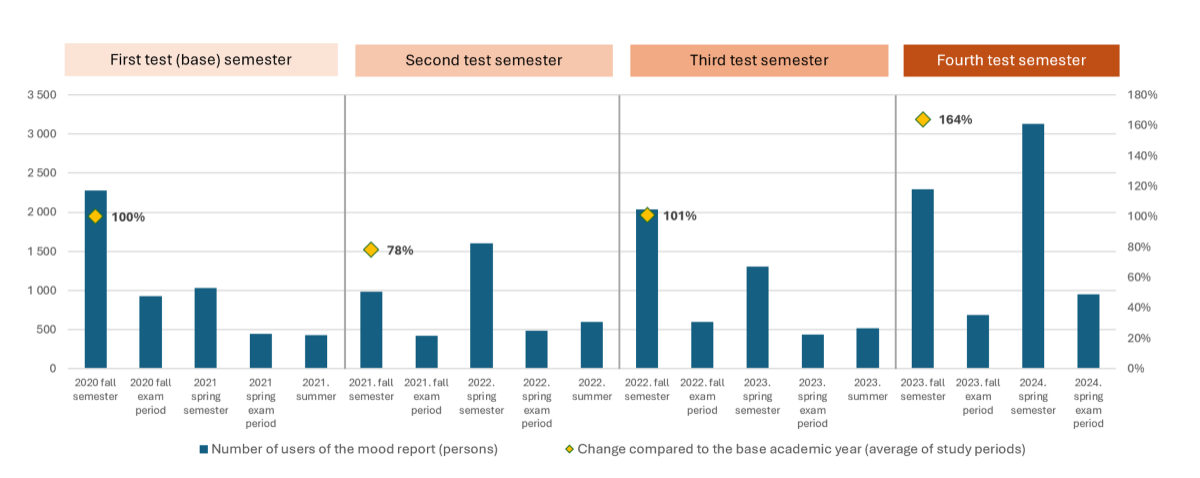
Number of students reporting their mood by academic study periods during the test period of the system.

In terms of the above-specified study periods, the UDSV and its mood reporting function were most frequently used during the semesters, and least used in the examination periods and during the summer vacation (summer; Figure 2).

**Figure 3:**
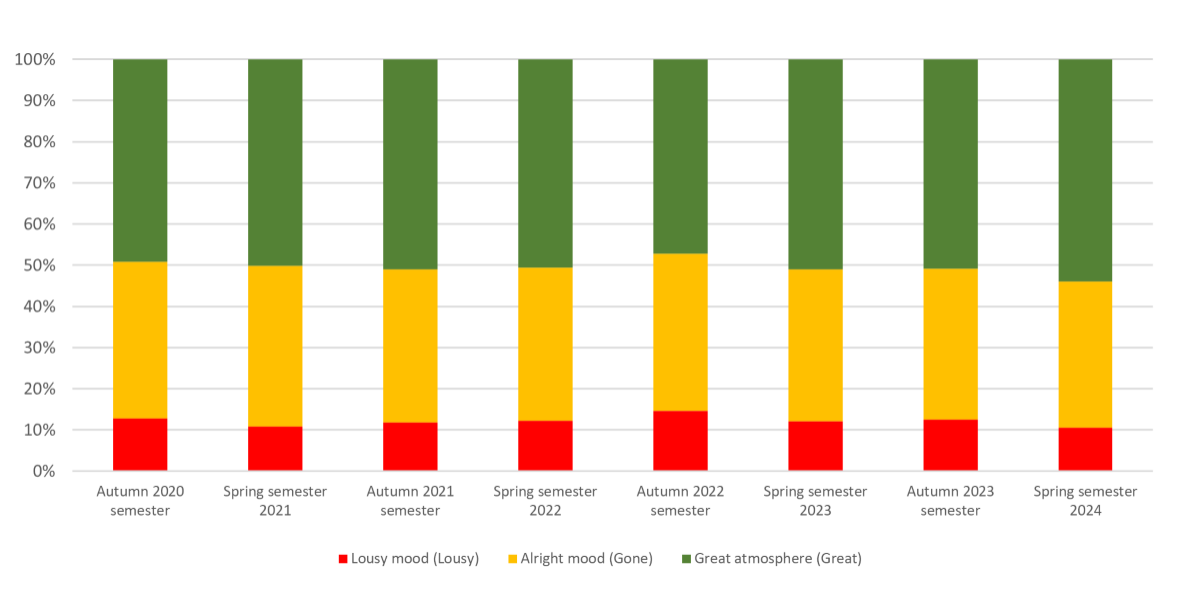
Distribution of the categories of mood reports by academic study periods during the test period of the system.

The distribution of the categories of the mood reports showed that the majority of students felt “alright” or “great”. The mean percent of “awful” reports was 11.6% (35,846/309,017 reports) during the full test period, 12.2% (27,379/224,418 reports) during the semesters, 12% (7158/59,650 reports) in the examination periods, and 8.9% (1309/14,708 reports) during the summer break. There was no significant difference in the proportion of “awful” mood reports during semesters and examination periods (*P*=.64; Figure 3).

## Discussion

This paper describes an integrated multilevel system newly developed by the University of Debrecen that provides mental help for its students, and reports on its 45-month test period between September 2020 and May 2024. There was a positive reception by students; approximately one-tenth of them used its mood report function at the first level of the system in the last half of the test period. More than 88% (261,957/297,678 reports) of students’ reports were acceptable (“alright”) or good (“great”) moods, and only approximately 12% (35,415/295,125 reports) were “awful” moods. Token rewards increased the number of users, as supported by the fact that from the launch of its introduction (February 2024), user numbers exceeded those of the previous years.

Based on 4 years of testing, the mood report embedded in the UDSV mobile app proved to be capable of identifying those students who are at an increased risk of having a mental health problem and are in need of support.

The benefits of using a mobile app include its all-time availability and easy reporting on their mood without stigmatization, whereas the university receives personalized and time-specific estimates on the mental health of students, which allows the identification of those at high risk of mental health problems and enables the provision of further screening based on which tailored suggestions for personal help can be delivered. A similar app assessing other functions can be used as a tool of needs assessment to organize and operate direct and efficient provision of health care in case of health emergencies, such as an epidemic.

Mobile phone penetration worldwide underwent an enormous, 1177-fold increase from 1999 to 2009 [42], and as of 2023, the global smartphone penetration rate was estimated at 69% [43]. Smartphone ownership at this mass scale enabled fundamental changes in the access and use of health care services with a shift to participatory direction [44]. Furthermore, 99% of Generation Z or Zoomers (youngsters, including college students, born after 1997) own a smartphone, and 98% of them use it to connect to the internet [45], so they are the most appropriate group to target with new information technologies.

As of now, evidence has already been available for the effectiveness of digital mental health interventions, most of them based on cognitive behavioural therapy [46] for university students for enhancing well-being [47] and improving the symptoms of depression and anxiety [30]. However, most of the studies were carried out as randomized experimental studies, not as campus-wide implementation studies. The majority of the interventions were delivered through a website, most commonly offering cognitive behavioral therapy with or without human support. Only a few studies examined the feasibility of campus-wide implementation. In one, students had to personally attend a clinic where they were screened, and if eligible, offered online treatment [48]. Another implementation study also recruited students from counseling centers that required personal attendance and screening, subsequent to which students could choose from one of three internet-delivered cognitive behavioral therapies [49]. App-based first-line screening seems to be a unique feature of our model. Its advantage is that its first means of encounter (the mood report) is offered to all students on a voluntary basis, and its use provides the information that enables the personalized invitation of those who report sustained bad mood.

The identification and recruitment of persons at increased risk of mental health problems remains a challenge, as has recently been pointed out by Fitzpatrick et al [50]. Extensive resources have been dedicated to developing digital mental health interventions, and though they represent scalable, low-cost solutions, their perceived lower desirability and credibility result in their underuse. This may be slightly different among students who showed interest in online self-help materials in varied forms and duration [51].

As to the future development of the system, including its mobile app, the authors of the most recent systematic review of smartphone apps made a number of recommendations that we agree with [34]. Among these are the multiplatform compatibility of apps, provision of feedback to clients and helpers, data protection, and involvement of users, including those with mental problems, during development. The greatest challenge is presented by the integration of smartphone apps with health care services, which is primarily ethical rather than operational. Respecting client autonomy and strict protocol for access to health care data for developers and system managers may or may not fully solve all these challenges.

One of the limitations of our system is related to the fact that two years (2020-2021) out of the full test period were markedly different from previous and subsequent years due to the pandemic that shifted most activities of the university to the online space, and also had an impact on students’ attention. This is reflected by the rather extensive swings in the number of mood reports and reporters between the first and the second half of the test period. The test period was quite long, so that usage well after the end of the pandemic could be assessed. Another limitation was due to the fact that the new app was promoted only two times: after its launch for 4 months, and in 2024 for 1 month, and these had a considerable impact on use. Still another limitation may be that students with preexisting mental conditions or having severe mental problems (eg, depression) may not use the app at all, for a lack of motivation or not having the willingness to report their mood in any form. A related additional challenge may be that students who use the application at Level 1 and are identified as at high risk of depression may not be willing to get in personal touch with helpers at Level 2, even if they are recommended to do so. This is in line with the underuse of health services by university students found by a large number of studies [27].

We developed an integrated multilevel model for improving the mental health of students at the University of Debrecen. We reported the testing of the first two digital levels of the model to assess the proportion of students for whom mental help should be offered. Campus-wide easy access and entry to the system were considered a basic condition, which required going beyond the traditional, face-to-face forms of mental health support. Therefore, a mood report function was developed and embedded into the general student support app of the university UD StudyVersity), which is available for all students on a smartphone to give self-reports in a nonstigmatizing manner. The mood report was proven to be capable of identifying students likely in need of further support. Approximately one-tenth of the students could be considered at high risk and will be invited for further screening and support. The development of digital services in a hierarchical manner that extends from an easy-to-use digital platform to other, more resource- and time-intensive services provided by trained professionals will be necessary to address the worldwide phenomenon of overwhelmed and distressed college students. However, such systems need continuous feedback, improvement, and promotion in order to reach all or most students for whom such a system would be of greatest use.

## Abbreviations

ACHE: Association for Counselling in Higher Education
BDI: Beck Depression Inventory
ESU: European Students’ Union
GHQ-12: 12-item General Health Questionnaire
MIS: Management Information System
OECD: Organization for Economic Co-operation and Development
OQ-45: Outcome Questionnaire–45
UDSV: UD Studyversity
WHO: World Health Organization

## Data Availability

The datasets analyzed in this study are not publicly available due to the primacy of protecting students’ privacy in line with Act CXII of 2012 of Hungary. Specific reasonable requests regarding the data should be sent to the first and last authors of this paper, who serve as the head manager of the system and chancellor, respectively.
